# Cytokine profile in first-episode drug-naïve major depressive disorder patients with or without anxiety

**DOI:** 10.1186/s12888-024-05536-2

**Published:** 2024-02-02

**Authors:** Jun Liang, Yayun Xu, Wenfan Gao, Yanhong Sun, Yuanyuan Zhang, Feng Shan, Qingrong Xia

**Affiliations:** 1 Affiliated Psychological Hospital of Anhui Medical University, Hefei, China; 2 Department of Pharmacy, Hefei Fourth People’s Hospital, Hefei, China; 3 Psychopharmacology Research Laboratory, Anhui Mental Health Center, Hefei, China; 4 Anhui Clinical Research Center for Mental Disorders, Hefei, China; 5 Department of Epidemiology and Biostatistics, School of Public Health, Anhui Medical University, Hefei, China; 6 Inflammation and Immune Mediated Diseases Laboratory of Anhui Province, Anhui Institute of Innovative Drugs, Anhui Medical University, Hefei, China; 7 The Key Laboratory of Anti-inflammatory and Immune Medicines, Ministry of Education, Hefei, China; 8 Department of Science and Education, Hefei Fourth People’s Hospital, Affiliated Psychological Hospital of Anhui Medical University, Anhui Mental Health Center, 316 Huangshan Road, 230000 Hefei, PR China

**Keywords:** Cytokines, Serum, Major depressive disorder, Anxiety, Correlation

## Abstract

**Objective:**

It is known that cytokines play a role in both depression and anxiety. This study aimed to compare the levels of multiple cytokines in patients with first-episode drug-naive major depressive disorder (MDD) with or without anxiety and analyze the correlation between the level of depression or anxiety and the serum cytokine levels.

**Methods:**

The study involved 55 patients with first-episode drug-naive MDD. To assess anxiety symptoms, the 14-item HAMA was used. MDD patients were divided into two groups: 23 MDD patients without anxiety and 32 MDD patients with anxiety. The measurement of 37 cytokines was conducted. Serum cytokine levels between patients with MDD without anxiety and anxiety were compared. In multiple linear regression models, the relationship between the group and abnormal cytokines was explored. The receiver operating characteristic (ROC) curve analysis was performed to estimate diagnostic performance of serum cytokines in discriminating MDD patients with anxiety from MDD patients without anxiety. A correlation was evaluated between the scores of HAMD or HAMA and the serum cytokine levels.

**Results:**

In MDD patients with anxiety, IL-17 C and CCL17 levels were significantly lower than in MDD patients without anxiety (all *P* < 0.05). Multiple measurements were corrected with Benjamini-Hochberger corrections, but none of these differences persisted (all *P* > 0.05). The results of multiple linear regression models revealed that after controlling for other independent variables, group was not a significant independent predictor of serum IL-17 C or CCL17 (all *P* > 0.05). The AUC values of IL-17 C and CCL17 were 0.643 and 0.637, respectively, in discriminating MDD patients with anxiety from MDD patients without anxiety. The results of partial correlation analyses showed the scores of HAMD were negatively correlated with the IL-17 C (*r* = -0.314, *P* = 0.021) levels with sex as a covariate.

**Conclusions:**

The findings suggest that there is a potential absence of disparity in the levels of circulating cytokines among individuals diagnosed with first-episode drug-naïve MDD, regardless of the presence or absence of comorbid anxiety.

**Supplementary Information:**

The online version contains supplementary material available at 10.1186/s12888-024-05536-2.

## Introduction

Major depressive disorder (MDD) is a serious and common mental disease characterized by depressed mood, anhedonia, cognitive impairment, and suicide tendencies [1]. Anxiety is one of the most prevalent comorbidities in MDD patients with a prevalence of 45–67% [2]. In comparison to MDD patients without anxiety, those with comorbid anxiety experience higher rates of suicide, more behavioral and psychiatric symptoms, longer illness duration, less antidepressant response, and fewer favorable outcomes [3–5]. Similar to MDD patients without anxiety, the majority of MDD patients with comorbid anxiety use antidepressants as their main treatment [6]. The underlying mechanism of comorbidity anxiety in patients with MDD is still unclear.

Multiple lines of evidence point to a close connection between MDD and cytokines. In a cumulative meta-analysis, it was shown that MDD patients had elevated peripheral levels of interleukin-6 (IL-6) [7]. Our previous study has indicated that the identification of biomarkers for the detection of suicidal ideation in patients with MDD may be aided by circulating IL-17 C and tumor necrosis factor-β (TNF-β) levels [8]. Additionally, alterations in peripheral cytokine levels in patients with MDD have been reported to be related to the outcomes of antidepressant treatment [9].

There is also a considerably strong correlation between anxiety and cytokines. It has been demonstrated that immune activation is linked to anxiety-like behavior and the increase of pro-inflammatory cytokines in peripheral circulation and brain in mice [10, 11]. Clinical data has suggested an association between anxiety symptoms and circulating cytokines, such as interferon-γ (IFN-γ) [12] and vascular endothelial growth factor (VEGF) [13].

Given that numerous evidence has demonstrated the relevant association between cytokines and MDD or anxiety, it is rational to hypothesize that there may be differences in serum cytokines between MDD patients without anxiety and MDD patients with anxiety. Few studies focused on the aberrant expression of cytokines in peripheral blood of patients with MDD with comorbid anxiety. It has been reported that cytokine profiles differ when comparing pregnant subjects exhibiting severe anxiety with comorbid severe depression against those showing only severe anxiety without depression [14]. In the current study, the serum levels of multiple cytokines in patients with first-episode drug-naïve MDD were detected, with the aim to discover serum cytokines that may involved in the mechanisms underlying comorbid anxiety in MDD. The 24-item Hamilton Rating Scale for Depression (HAMD) and the 14-item Hamilton Anxiety Rating Scale (HAMA) were used to assess the symptoms of depression and anxiety, respectively. Subsequently, the levels of 37 serum cytokines between MDD patients without anxiety and MDD patients with anxiety were compared. The relationship between the scores of HAMD or HAMA and the concentrations of serum cytokines was assessed.

## Methods

### Study design and participants

The study was conducted from August 2020 to June 2022 at Anhui Mental Health Center. All patients with first-episode drug-naïve MDD were diagnosed by trained psychiatrists according to Diagnostic Criteria of American Diagnostic and Statistical Manual of Mental Disorders Fifth Edition (DSM-5). All MDD patients were inpatients and met the following inclusion criteria: (1) age 18–65; (2) HAMD scores higher than 20; (3) not taking any psychotropic drugs, anti-inflammatory medications, or antidepressants in the past three months. Exclusion criteria included: (1) neurological or other psychiatric disorders; (2) substance abuse/dependence (other than tobacco); (3) chronic inflammatory diseases. The size of the study sample was estimated using the G*Power 3.1.9 program. The power analysis was conducted using an alpha of 0.05, a power (1-β) of 0.80, and a medium effect size of 0.50 for a two-tailed test. The desired total sample size for detecting differences between two groups was 128. However, owing to the restricted pool of individuals diagnosed with first-episode drug-naïve MDD over a span of two years, coupled with the substantial expenses associated with conducting tests, only 55 MDD patients were enrolled in the present study. This procedure was approved by the ethics committee of the Anhui Mental Health Center (registration number HFSY-IRB-PJ-XQR-2,020,001) and was conducted according to the principles of the Declaration of Helsinki. Informed consent was obtained from all the participants.

### Clinical assessment

The 14-item HAMA was used to estimate the symptoms of anxiety. In the 14-item HAMA scale, each item is scored on a scale of 0 (not present) to 4 (severe), with a total score range of 0 to 56, where 0 represents no, < 17 represents mild, 18 to 24 represents moderate, 25 to 30 represents severe, and > 30 represents very severe anxiety. A cut-off point of 18 was used to divide patients into groups with or without anxiety symptoms [15]. Fifty-five MDD patients were divided into two groups accordingly: 23 MDD patients without anxiety and 32 MDD patients with anxiety.

Suicidal ideation (SI) was estimated by the 19-item Beck Scale for Suicide Ideation [16]. Individuals who scored > 0 on either item 4 or 5 were considered to be currently suicidal. Individuals who received a score of 0 for both these items were considered to be currently non-suicidal [17].

### Collection of blood sample and measurement of serum cytokines

Venous blood samples were collected between 7:00 and 8:00 AM into test tubes without any anticoagulant. For serum separation, whole blood was allowed to clot for 30 min at room temperature, and then centrifuged at 1200 g for 10 min at 4 °C. The supernatant serum samples were stored immediately at -80 °C until detection. In order to control the effect of drugs, blood samples were collected at baseline before treatment. A total of 37 serum cytokines were measured by the multiplex bead immunoassay (LXSAHM-10 and LXSAHM-27, R&D system for antibody detection, Shanghai Universal Biotech Co., Ltd) including internal quality control material with an automated analyser (Luminex 200, Luminex Corporation, Austin, USA) according to the manufacturer’s instructions. Standard curves were generated by using the reference cytokine sample supplied in the kit and were used to calculate the cytokine concentrations in aqueous humor samples. Except for a few values below the detection limit, all other values were within the calibration curve interval.

### Statistical analysis

SPSS (version 17.0; IBM Corp., Armonk, NY, USA) was applied for statistical analysis. The level of statistical significance was set at *P*-value < 0.05. The normality of continuous variable was evaluated using the Kolmogorov-Smirnov normality test. Normally distributed data were compared using the Student’s t-test, whereas data following a non-normal distribution were analyzed using a non-parametric test (Mann-Whitney U test). Normally distributed quantitative variables presented as mean ± standard error of the mean (SEM), and skewed quantitative variables presented as median and interquartile range (IQR; 25–75%). The Benjamini-Hochberg (BH)-corrected *P*-value (FDR-False Discovery Rate) was calculated to adjust for multiplicity of testing. Sex, marital status, and smoking status between the two groups were compared by chi-square test. The relationship between the group and the abnormal cytokines were investigated in multiple linear regression models, with adjustments for age, gender, BMI, education, marital status, smoking status, and HAMD-24 scores. The receiver operating characteristic (ROC) curve analysis was performed to estimate diagnostic performance of serum cytokines in discriminating MDD patients with anxiety from MDD patients without anxiety. Correlations analyses were performed using Pearson correlation on normally distributed data and Spearman’s correlation for non-normally distributed data. Additionally, in order to exclude the influence of gender on these correlations, partial correlation analyses were conducted with sex as a covariate. To ensure that the results were not unduly influenced by a single data point and as is the convention, outliers (datapoint > 3 SD above or below the mean) were removed from the dataset.

## Results

### Demographic and clinical characteristics of the patients

The demographic and clinical characteristics of MDD patients without anxiety and MDD patients with anxiety are summarized in Table 1. No significant differences in age, BMI, years of education, marital status, smoking status, or suicidal ideation were found between the two groups (all *P* > 0.05). There were significant differences in gender ratio between the two groups (*P* < 0.05). The prevalence of comorbid anxiety disorders was 68.6% and 40.0% in female and male MDD patients, respectively. The HAMD-24 scores of MDD patients with anxiety were significantly higher than those of MDD patients without anxiety (*P* < 0.05).

**Table 1:** Demographic and clinical characteristics of MDD patients without anxiety and MDD patients with anxiety

Variables	MDD without anxiety	MDD with anxiety	t/χ^2^	*P*
Age	36.13 ± 2.75	36.31 ± 2.74	-0.046	0.964
Sex (Female/Male)	11/12	24/8	4.270	0.039
BMI (kg/m^2^)	22.61 ± 0.64	22.34 ± 0.65	0.284	0.778
Education (years)	10.13 ± 0.98	9.12 ± 0.95	0.719	0.475
Marital status (Yes/No)	12/11	19/13	0.282	0.595
Smoking status (Yes/No)	4/19	3/29	0.774	0.379
Suicidal ideation (Yes/No)	13/10	14/18	0.873	0.350
HAMD-24	31.87 ± 1.74	37.37 ± 1.73	-2.189	0.033
HAMA	11.87 ± 0.71	26.26 ± 1.18	-10.431	< 0.001

Normally distributed quantitative variables presented as mean ± standard error of the mean (SEM)

### Comparison of serum cytokine levels between MDD patients without anxiety and MDD patients with anxiety

**Fig. 1:**
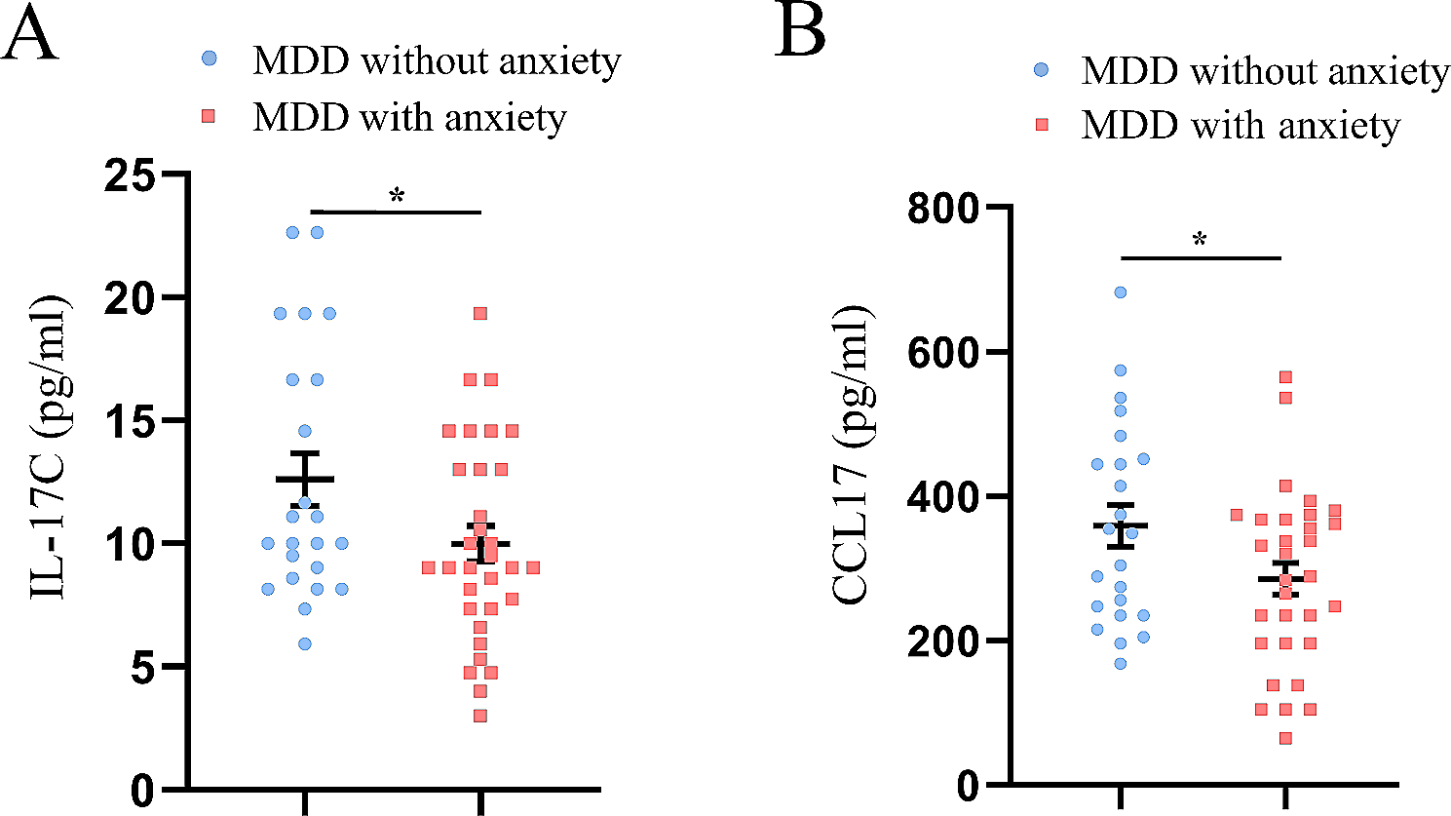
Serum levels of different cytokines in MDD patients without anxiety and MDD patients with anxiety. (**A**) Comparison of IL-17 C; (**B**) Comparison of CCL17. The data are presented as the mean ± SEM. *P < 0.05

As shown in Table 2; Fig. 1, the levels of IL-17 C and CCL17 in MDD patients with anxiety were significantly lower than those in MDD patients without anxiety (all *P* < 0.05). Multiple measurements were corrected with Benjamini-Hochberger corrections, but none of these differences persisted (all *P* > 0.05). There were no significant differences in other cytokines levels between the two groups (Table 2).

**Table 2:** Comparison of serum cytokines between MDD patients without anxiety and MDD patients with anxiety

Variables (pg/ml)	MDD without anxiety	MDD with anxiety	t/Z	*P*	BH adj. *P*-value
IL-1α (IL-1F1)	2.24 (1.42, 4.30)	2.74 (1.81, 4.68)	-0.887	0.375	1.156
IL-1β (IL-1F2)	6.79 (6.34, 10.39)	6.34 (4.77, 8.23)	-0.842	0.400	1.057
IL-1RA (IL-1F3)	956.58 ± 90.51	917.24 ± 79.09	0.323	0.748	0.954
IL-2	31.63 ± 4.86	27.24 ± 4.99	0.795	0.430	0.936
IL-3	26.53 (23.86, 26.53)	26.53 (23.86, 29.36)	-0.486	0.627	0.859
IL-4	27.82 (9.99, 60.43)	23.17 (6.95, 37.86)	-0.980	0.327	1.210
IL-5	2.04 (1.45, 2.41)	2.04 (1.45, 2.04)	-0.157	0.875	0.981
IL-6	5.43 (1.75, 12.42)	3.43 (2.20 12.83)	-0.029	0.977	0.977
IL-7	10.90 ± 1.29	9.98 ± 1.07	0.551	0.584	0.864
IL-8 (CXCL8)	513.66 ± 102.12	428.50 ± 72.05	0.702	0.486	0.856
IL-10	2.80 (0.97, 6.22)	1.54 (1.54, 2.80)	-1.317	0.118	1.092
IL-12 (IL-23 p40)	226.13 ± 10.62	221.69 ± 11.16	0.276	0.784	0.907
IL-12 p70	25.63 ± 2.86	23.33 ± 2.39	0.620	0.538	0.829
IL-13	243.20 ± 11.21	242.06 ± 7.85	0.086	0.932	0.958
IL-15	2.86 (1.54, 4.82)	3.60 (2.34 6.12)	-0.983	0.326	1.340
IL-16	178.42 ± 16.88	172.52 ± 11.49	0.300	0.766	0.914
IL-17 C	12.61 ± 1.07	10.00 ± 0.72	2.111	0.040	1.480
IL-27	296.95 ± 20.63	301.21 ± 24.85	-0.127	0.900	0.951
IL-31	26.20 (21.40, 31.81)	26.20 (21.40, 31.81)	-0.130	0.897	0.976
CCL3 (MIP-1α)	608.56 ± 111.68	515.78 ± 71.89	0.737	0.465	0.906
CCL4 (MIP-1β)	445.93 ± 69.59	421.01 ± 44.93	0.315	0.754	0.930
CCL11 (Eotaxin)	127.97 ± 12.44	106.11 ± 8.07	1.539	0.130	0.802
CCL17 (TRAC)	359.01 ± 28.98	285.69 ± 21.99	2.054	0.045	0.833
CCL26 (Eotaxin-3)	14.91 ± 1.24	13.66 ± 0.98	0.798	0.429	0.992
CXCL10 (IP-10/CRG-2)	18.66 ± 1.19	19.59 ± 1.18	-0.538	0.593	0.844
VEGF	110.95 ± 12.61	89.95 ± 6.62	1.595	0.117	1.443
VEGF-C	1644.09 ± 117.19	1778.98 ± 97.67	-0.883	0.381	1.084
VEGFR1 (Flt1)	163.04 ± 12.86	147.05 ± 9.11	1.044	0.301	1.591
TNF-α	4.46 (2.87, 6.57)	3.33 (2.87, 5.34)	-0.722	0.471	0.871
TNF-β (Lymphotoxin)	2.76 ± 0.21	2.58 ± 0.15	0.683	0.498	0.838
Tie-2	16038.61 ± 1342.56	14912.87 ± 1150.79	0.638	0.526	0.846
IFN-γ	14.92 (11.85, 23.83)	14.92 (11.85 18.40)	-0.834	0.404	0.997
GM-CSF	3.94 ± 0.41	3.21 ± 0.26	1.569	0.123	0.910
FGF basic (FGF2/bFGF)	10.25 ± 1.46	9.53 ± 1.17	0.387	0.700	0.925
TSLP	1.14 ± 0.07	1.06 ± 0.05	1.032	0.307	1.420
ICAM-1 (CD54)	233,586 (71,458, 561,730)	236,650 (186,478, 599,410)	-0.922	0.357	1.201
PIGF	2.16 ± 0.16	2.33 ± 0.14	-0.781	0.438	0.900

BH adj, Benjamini-Hochberg adjusted *P*-value. Normally distributed quantitative variables presented as mean ± standard error of the mean (SEM), and skewed quantitative variables presented as median and interquartile range (IQR; 25–75%)

### Multiple linear regression analyses were used to assess the association between the group and abnormal cytokines

The relationship between the group and the abnormal cytokines was investigated in multiple linear regression models, with adjustments for age, gender, BMI, education, marital status, smoking status, and HAMD-24 scores. The results revealed that after controlling for other independent variables, group was not a significant independent predictor of serum IL-17 C (*β* = -0.169, *t* = -1.168, *P* = 0.249) or CCL17 (*β* = -0.198, *t* = -1.390, *P* = 0.171).

### Diagnostic performance of different cytokines in discriminating MDD patients with anxiety from MDD patients without anxiety

**Fig. 2:**
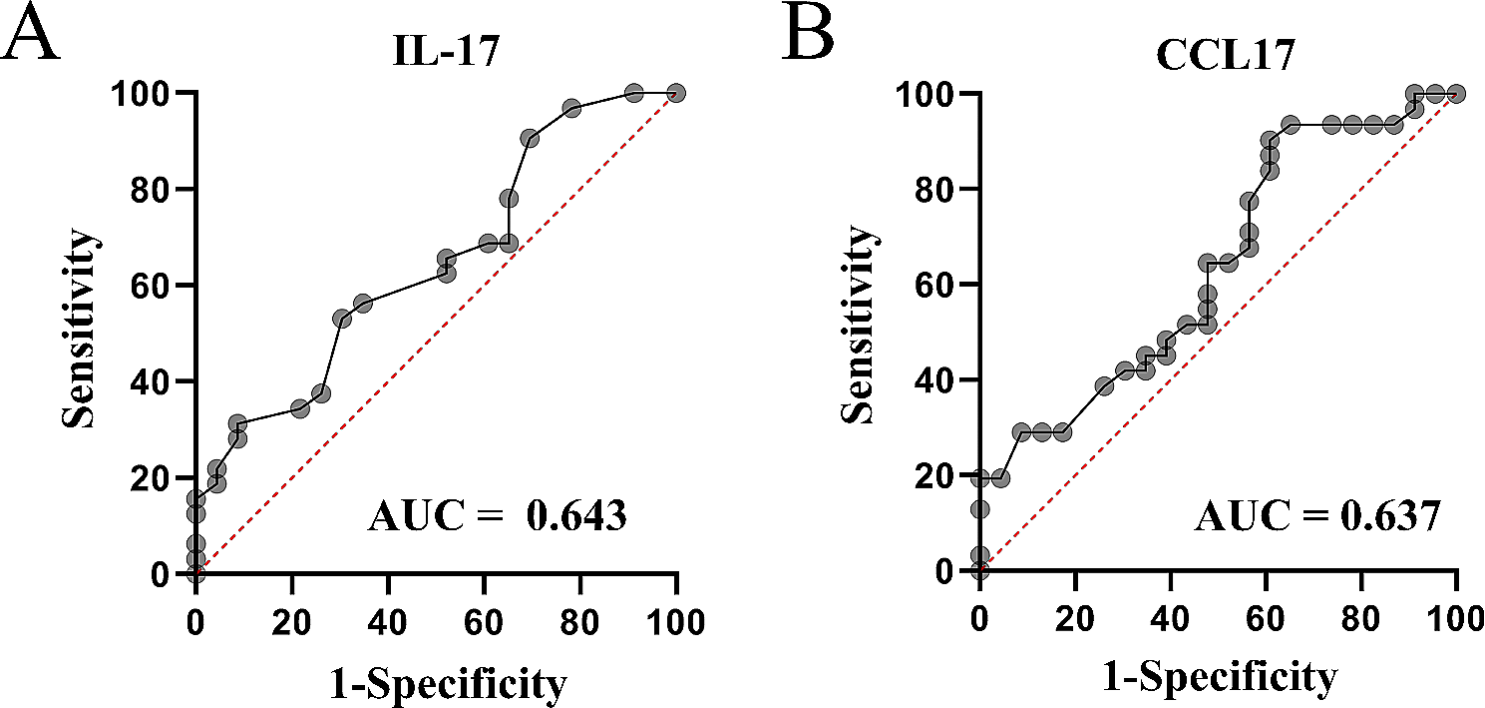
ROC curves of IL-17 C and CCL17 in discriminating MDD patients without anxiety from MDD patients with anxiety. (**A**) ROC curve of IL-17 C; (**B**) ROC curve of CCL17

A ROC curve analysis was used to assess the performance of different cytokines in distinguishing MDD patients with anxiety from MDD patients without anxiety (Fig. 2). The area under the curve (AUC) values of IL-17 C and CCL17 were 0.643 and 0.637, respectively. As a general rule, AUC in ROC analysis should be greater than 0.7 to be considered clinically useful. Thus, IL-17 C and CCL17 have inadequate accuracy in discriminating MDD patients without anxiety from MDD patients with anxiety.

### Correlation between the HAMD or HAMA scores and the cytokine levels in serum

Figure S1 showed that the HAMD scores were negatively correlated with the IL-17 C (*r* = -0.300, *P* = 0.026) levels. Moreover, the HAMA scores were negatively correlated with the IL-17 C (*r* = -0.270, *P* = 0.048) and CCL17 (*r* = -0.286, *P* = 0.038) levels, and positively correlated with the IL-12 p70 (*r* = 0.299, *P* = 0.037) levels. In order to exclude the influence of gender on these correlations, partial correlation analyses were conducted with sex as a covariate. The results showed that no significant relationship was found between the HAMA scores and the levels of serum IL-17 C (*r* = -0.224, *P* = 0.107), CCL17 (*r* = -0.218, *P* = 0.121), or IL-12 p70 (*r* = 0.059, *P* = 0.694). There was still a negative relationship between the HAMD scores and the levels of IL-17 C (*r* = -0.314, *P* = 0.021).

## Discussion

The present study investigated the serum levels of multiple cytokines in patients with first-episode drug-naïve MDD, with the aim to discover serum cytokines that may involved in the mechanisms underlying comorbid anxiety in MDD. Three main findings emerged from the present study. First, there were no significant differences in cytokines levels between the two groups. Second, the scores of HAMD were negatively correlated with the IL-17 C levels. Third, no significant relationship was found between the HAMA scores and the levels of cytokines.

Cytokines are reported to play crucial roles in the occurrence and development of psychological disorders including depression and anxiety. However, the underlying mechanism is not fully understood, and it may be related with the following aspects: (1) cytokines regulate intracellular metabolic processes to promote the oxidative stress and neural apoptosis, and cause anxiety and depression [18]; (2) the signal integrity of neurotransmitters in the cerebral cortex and hippocampus can be compromised by cytokines, which can lead to an imbalance of neurotransmitters and psychiatric disorders including depression and anxiety [19]; (3) cytokines can induce the activation of neuroglia, which has been acknowledged as a triggering factor for psychiatric disorders [20]. Therefore, establishment of abnormal expression profile of cytokines in peripheral blood in patients with MDD with or without anxiety may provide a new way to explore the underlying mechanism of comorbidity anxiety in patients with MDD.

IL-12p70, the biologically active form of IL-12, is composed of two subunits: the constitutively expressed IL-12p35 and the inducible IL-12p40. IL-12p70 is one of the first pro-inflammatory cytokines released after antigens from the foreign agents [21]. Previous studies have demonstrated that IL-12p70 is critical for the immune response to tumors, intracellular parasites, fungi, bacteria, and viruses [22, 23]. To the best of knowledge, this is the first study demonstrating a potential positive correlation between the serum IL-12 p70 levels and the level of anxiety in patients with MDD. However, no significant relationship between the HAMA scores and the levels of serum IL-12 p70 was found when controlling for sex as a covariate. Since this study is a single-center study and the sample size is relatively small, this correlation should be confirmed by multicentric studies.

IL-17 C is a poorly studied member of the IL-17 cytokine family. Its synthesis is induced by various cell types of cytokines and pathogenic stimuli [24]. Previous studies have shown that IL-17 C plays a key role in the pathogenesis of various autoimmune diseases and autoimmune diseases [25]. Our recent study has found that compared with MDD patients without suicidal ideation, the serum IL-17 C concentration of MDD patients with suicidal ideation was significantly higher [8]. Moreover, the AUC of IL-17 C was 0.728 in distinguishing MDD patients with suicidal ideation from MDD patients without suicidal ideation, with a sensitivity of 86.2% and a specificity of 58.3% [8]. In the present study, no significant differences in IL-17 C levels between MDD patients without anxiety and MDD patients with anxiety. Moreover, serum IL-17 C levels were found to be negatively correlated with the HAMD scores, but not the HAMA scores. Given that there is a strong relationship between severity of depressive symptoms and suicidal ideation, the finding that serum IL-17 C levels exhibit a negative correlation with the severity of depressive symptoms, while concurrently increasing in individuals diagnosed with MDD and experiencing suicidal ideation, presents a contradictory observation. The precise mechanisms underlying these conflicting findings remain unclear, necessitating further investigation to elucidate.

CCL17 is a member of the CC chemokine group that is constitutively expressed in the thymus and is produced by dendritic cells, endothelial cells, keratinocytes, bronchial epithelial cells and fibroblasts [26]. More recently, it has been demonstrated that CCL17 levels were positively correlated with depression and anxiety levels in patients with asthma [27]. However, another study have shown that the cerebrospinal fluid levels of CCL17 were significantly decreased in patients who attempted suicide compared to with healthy control subjects [28]. Consistently, our previous study has reported that patients with MDD had lower levels of serum CCL17 compared to controls, and ROC curve analysis showed that the AUC value of CCL17 was > 0.7 in discriminating patients with MDD from healthy controls [29]. In the present study, following the Benjamini-Hochberg correction for multiple comparisons, there were no significant differences in CCL17 levels between the two groups. No significant relationship was found between the HAMA scores and the levels of CCL17 when controlling for sex as a covariate. Therefore, these results suggest that peripheral CCL17 may play a role in the underlying mechanisms of MDD, but not in the presence of comorbid anxiety.

In a study conducted across 15 psychiatric hospitals and clinics, it was found that 68.9% of the 508 patients diagnosed with MDD experienced comorbid anxiety disorders [30]. Similarly, the prevalence of comorbid anxiety disorders in female and male MDD patients was determined to be 68.6% and 40.0%, respectively, in the current investigation. These findings contribute further evidence to support the coexistence of depression and anxiety. Recent research has indicated that individuals diagnosed with anxious depression displayed more pronounced symptoms of depression [31, 32]. We also revealed that patients diagnosed with MDD and comorbid anxiety exhibited significantly elevated HAMD-24 scores compared to those diagnosed with MDD alone. Considering that the presence of comorbid anxiety is linked to a diminished response to antidepressant medication, a lower rate of achieving remission, a higher probability of experiencing adverse effects during treatment, and an elevated risk of relapse in patients with MDD [33], the evaluation of anxiety-related attributes in patients diagnosed with MDD holds significance in informing the development of effective treatment approaches. Despite the absence of discernible disparities in cytokine levels among the two cohorts, investigating the prospective involvement of cytokines in the co-occurrence of anxiety disorder and depression via extensive and multi-center investigations could serve as a promising avenue for future research.

Several limitations of the present study should be highlighted. Firstly, the relatively small sample size of this single-center study could not be ignored. Secondly, this is a cross-sectional study so we are unable to establish a causal relationship between these cytokines and anxiety in MDD patients. Thirdly, the lack of a normal control group is also another limitation to this study.

## Conclusion

In conclusion, the present study for the first time to compare the levels of multiple cytokines in patients with first-episode drug-naive MDD with or without anxiety and analyze the correlation between the level of depression or anxiety and the serum cytokine levels. The results indicate a potential lack of discrepancy in the concentrations of circulating cytokines among individuals who have been diagnosed with first-episode drug-naïve MDD, irrespective of the coexistence or nonexistence of comorbid anxiety.

### Electronic supplementary material

Below is the link to the electronic supplementary material.

Supplementary Material 1

## Data Availability

The datasets generated during and/or analyzed during the current study are available from the corresponding author on reasonable request.
