# Supplementary material for: Cytokine profile in first-episode drug-naïve major depressive disorder patients with or without anxiety
Source: BMC Psychiatry. 2024 Feb 2;24:93. doi: 10.1186/s12888-024-05536-2 (PMC10835958; doi:10.1186/s12888-024-05536-2)

**Figure S1** Correlation between the HAMD or HAMA scores and the cytokine levels in serum in MDD patients. ×: no significance.


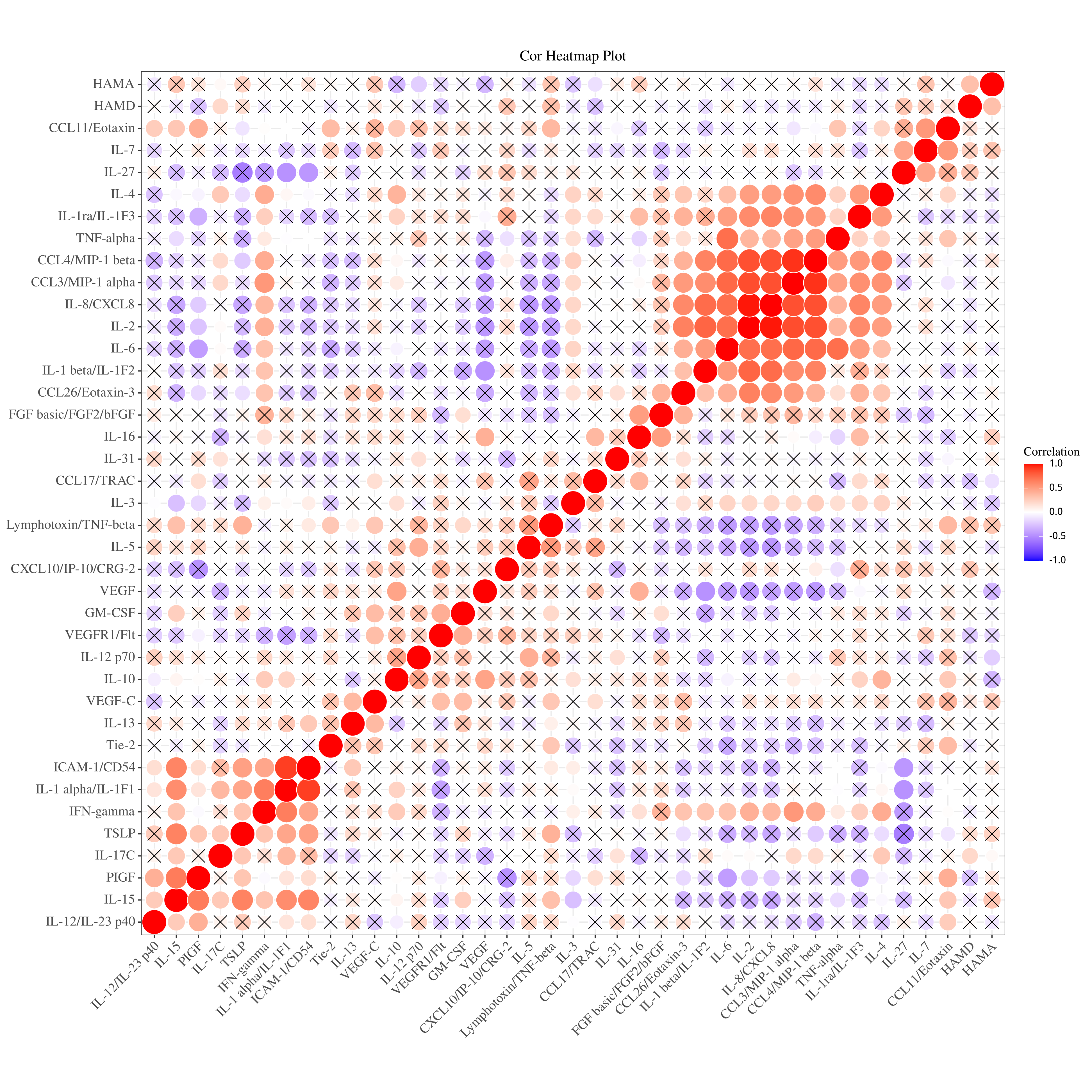

Supplement: Supplementary file 1 — Supplementary Material 1 [file 12888_2024_5536_MOESM1_ESM.docx]
